# Effectiveness of a digital device providing real-time visualized tooth brushing instructions: A randomized controlled trial

**DOI:** 10.1371/journal.pone.0235194

**Published:** 2020-06-25

**Authors:** Haruka Shida, Satoe Okabayashi, Masami Yoshioka, Naoko Takase, Masahiro Nishiura, Yui Okazawa, Kosuke Kiyohara, Manako Konda, Norihiro Nishioka, Takashi Kawamura, Taku Iwami

**Affiliations:** 1 Department of Preventive Services, Kyoto University School of Public Health, Kyoto, Japan; 2 Kyoto University Health Service, Kyoto, Japan; 3 Department of Oral Health Sciences, Faculty of Health and Welfare, Tokushima Bunri University, Tokushima, Japan; 4 R&D Department, Sunstar Inc., Osaka, Japan; 5 Department of Food Science, Otsuma Women’s University, Tokyo, Japan; Harvard School of Dental Medicine, UNITED STATES

## Abstract

**Introduction:**

The aim of this trial was to investigate whether a digital device that provides real-time visualized brushing instructions would contribute to the removal of dental plaque over usual brushing instructions.

**Methods:**

We conducted a single-center, parallel-group, stratified permuted block randomized control trial with 1:1 allocation ratio. Eligibility criteria included people aged ≥ 18 years, and exclude people who met the following criteria: severely crowded teeth; using interdental cleaning implement; having external injury in the oral cavity, or stomatitis; having less than 20 teeth; using orthodontic apparatus; visited to a dental clinic; having the possibility of consulting a dental clinic; having a dental license; not owning a smartphone or tablet device; smoker; taken antibiotics; pregnant; an allergy to the staining fluid; and employee of Sunstar Inc. All participants received tooth brushing instructions using video materials and were randomly assigned to one of two groups for four weeks: (1) an intervention group who used the digital device, providing real-time visualized instructions by connection with a mobile application; and (2) a control group that used a digital device which only collected their brushing logs. The primary outcome was the change in 6-point method plaque control record (PCR) score of all teeth between baseline and week 4. The *t*-test was used to compare the two groups in accordance with intention-to-treat principles.

**Results:**

Among 118 enrolled individuals, 112 participants were eligible for our analyses. The mean of PCR score at week 4 was 45.05% in the intervention group and 49.65% in the control group, and the change of PCR score from baseline was −20.46% in the intervention group and −15.77% in the control group (*p* = 0.088, 95% confidence interval −0.70–10.07).

**Conclusions:**

A digital device providing real-time visualized brushing instructions may be effective for the removal of dental plaque.

## Introduction

Periodontal disease is an inflammatory condition caused by infection with bacteria and is one of the main diseases seen in dentistry [1]. Decayed teeth and periodontal disease represent the two majors causes of tooth loss [1]. The national survey of dental disease in Japan showed that the proportion of the people who have periodontal pockets over 4 mm was 25.7–31.4% of people aged in the twenties and 47.8–54.1% aged in the fifties [2]. Moreover, this proportion has been increasing in the five years from 2011 to 2016 [1,2].

Once a tooth is lost from periodontal disease, it causes oral dysfunction and influences the whole body [1]. Many previous studies have investigated the association between periodontal disease and other conditions, especially lifestyle-related diseases such as atherosclerosis and diabetes mellitus [3–6]. An association between pneumonia in old age and poor oral hygiene has been reported [7]. The prevention and treatment of periodontal disease has become an important societal and public health issue.

Removing dental plaque is important for the prevention and treatment of periodontal disease [1]. The easiest self-care is through using a toothbrush and other dental materials. Previous studies have shown the effectiveness of removing plaque by brushing or brushing instructions [8–12]. Further, guidelines for periodontal disease treatment emphasize the importance of four processes in plaque control: motivation; self-care; the procedure of brushing; and professional care [1].

Appropriate brushing instructions are required to master effective brushing. However, not many people visit the dentist regularly, which is of note as people can get brushing instructions from dentists or dental hygienists. Moreover, one of the tooth brushing methods which is often used in the clinical setting is the Bass method, which requires one to move their toothbrush in small steps [9,13]. Previous studies have suggested that it is difficult for people to master appropriate tooth brushing methods and skills [10,11].

In addition, although toothbrushing for more than two minutes is recommended to remove dental plaque [14], a study showed that only around 20% of the population satisfy the appropriate tooth-brushing time [15]. One of the reasons for this may be the difficulties in regularly brushing teeth for an appropriate amount of time in the presence of a busy lifestyle.

Sunstar Inc. (Osaka, Japan) has developed a digital device that provides visible brushing instructions, linked to a mobile application, to facilitate learning appropriate teeth brushing habits. Many similar applications and devices, which improve oral hygiene or promote oral health behavior, have been developed and there have been reported their effect of only mobile applications or especially with electric toothbrush [16–21]. However, little is known about their effect with manual toothbrush, and the association between visualized brushing instructions and plaque control or the habituation of appropriate brushing methods [22–25]. The aim of this trial was to investigate whether brushing teeth manually with the digital device, which provides real-time visualized brushing instructions, was superior in the removal of dental plaque and habituation of appropriate brushing techniques compared to usual brushing instructions.

## Methods

### Trial design

We conducted a single-center, parallel-group, randomized control trial (RCT). Randomization was done by stratified permuted block with a 1:1 allocation ratio.

### Participants, eligible criteria, and settings

The trial was conducted at Kyoto University Health Service from October 29^th^, 2018 to November 30^th^, 2018. We placed an advertisement about this trial in Kyoto University’s bulletin board from October 3rd, 2018 and recruited participants from October 29^th^ to November 2nd. At the first day of follow-up period, after explaining this trial and gave written informed consents, we registered the participant officially, and followed each participant for one month.

This trial included people aged ≥ 18 years, and we excluded people who met one or more of the following criteria: severely crowded teeth (one third or more of the teeth overlapping, making it difficult to remove the dental plaque using a toothbrush alone); using interdental cleaning implement every day; the presence of an external injury in the oral cavity, or stomatitis, that can affect teeth brushing; having less than 20 remaining teeth; using orthodontic apparatus; visit to a dental clinic within the past month; having the possibility of consulting a dental clinic during the study period; having a dental license; not owning a smartphone or tablet device; current smoker; taken antibiotics within the past week; the possibility of being pregnant; an allergy to the staining fluid used for dental plaques; and being an employee of Sunstar Inc.

The trial was approved by the ethics committee of Kyoto University (C1390) on June 27^th^, 2018, and registered University hospital Medical Information Network (UMIN) Clinical Trial Registry (CTR) (UMIN000034503).

### Procedure

Before the trial, participants were measured plaque control record (PCR) score and underwent brushing for dental plaque removal by a dental hygienist. The PCR score was measured by O’Leary’s method [26], where the tooth surface is divided into 4 parts (buccal, lingual, medial, distal) and the presence or absence of plaque in each part is evaluated. In this trial, we also used the 6-point method, which involves dividing the tooth surface into 6 parts (buccal medial, buccal center, buccal distal, lingual medial, lingual center, lingual distal) to provide a more detailed report. The PCR scores of the 4-point and 6-point methods were calculated as follows:

$$\frac{\text{number}\;\text{of}\;\text{surfaces}\;\text{with}\;\text{plaque}}{\text{total}\;\text{number}\;\text{of}\;\text{surfaces}}\times 100[\%]$$

**Fig 1:**
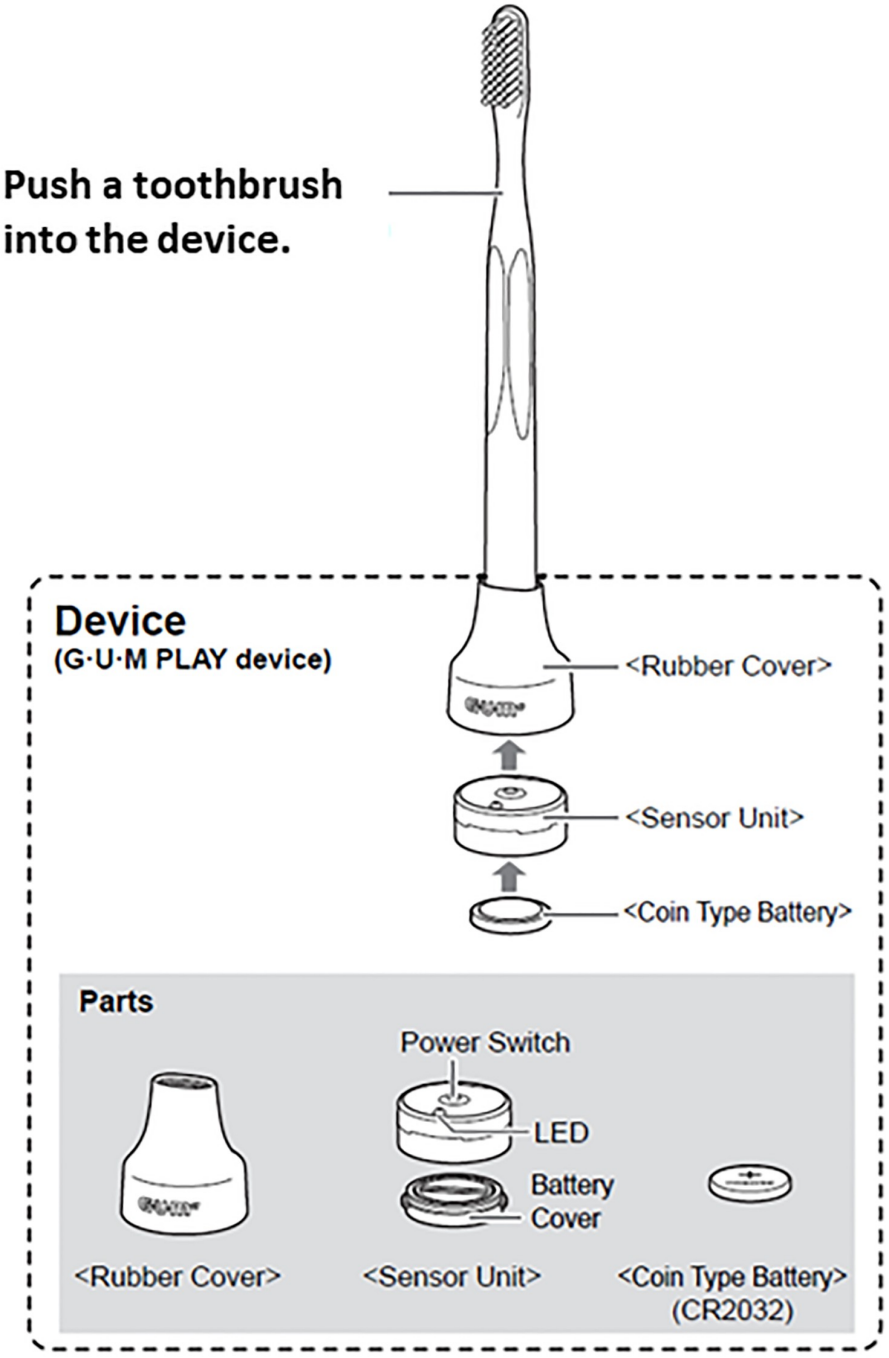
GUMPLAY^®^ device.

**Fig 2:**
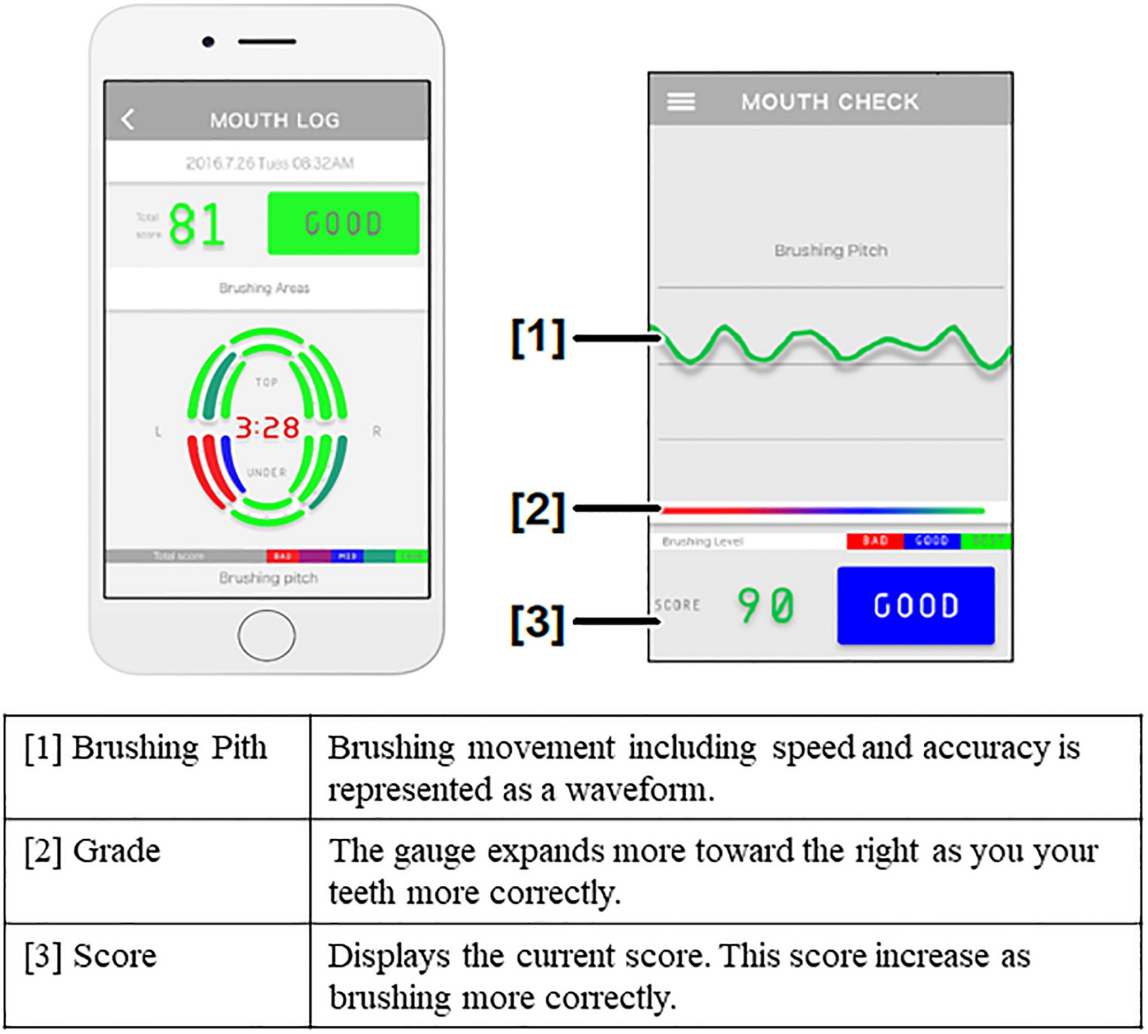
Image of brushing instructions that be shown on the screen of smartphone or tablet.

Subsequently, all participants received tooth brushing instructions using video teaching materials and were then randomly assigned to one of the two groups. The participants in the intervention group brushed their teeth for four weeks using the GUMPLAY^®^ device, which had three functions linked with a mobile application: (1) to visualize the toothbrush position and provide a guide for the brushing sequence; (2) to ensure the brushing time lasted more than 3 minutes; and (3) to visualize whether the user is moving the toothbrush in small steps or not (Figs 1 and 2). The participants of the control group brushed their teeth as usual for four weeks using GUMPLAY^®^, which was only used to collect their brushing logs. The GUMPLAY^®^ in each group was installed at the bottom of the toothbrush.

All participants used a standard toothbrush (GUM dental brush #166M, Sunstar Inc., Osaka, Japan) and toothpaste (Ora^2^ me STAIN CLEAR, Sunstar Inc., Osaka, Japan) during the trial and were restricted from using other dental materials for plaque removal, such as dental floss and mouthwash. The PCR score was measured 2 and 4 weeks after the baseline measurement by the dental hygienist. The participants were prohibited from eating, drinking and tooth brushing two hours before the oral examination, so that the time and the oral conditions at the three examinations were as similar as possible.

### Outcomes

The primary outcome was the change in the 6-point method PCR score of all teeth between baseline and week 4. The secondary outcome was the change in the 4-point method PCR score of all teeth between baseline and week 4. We also evaluated the consciousness of the brushing procedure, brushing each surface of the teeth, moving the toothbrush in small steps, and the time spent for brushing in one episode, and free comments at baseline and at week 4, using questionnaire survey.

The PCR score was calculated by three dental hygienists using staining fluid. The same dental hygienist measured the same participant at the three measurement times, and calibration for minimalizing bias among dental hygienists was enacted by providing education beforehand.

### Sample size

The sample size of this trial was calculated based on the results of a pilot study conducted by Sunstar Inc. In the pilot study, the participants were nine adults aged ≥18 years old who were observed for two weeks. The PCR was measured (A) followed by brushing instructions from a dental hygienist. A week later, PCR was re-measured (B). On the same day, the digital device provided visualized brushing instructions was supplied to the participants, and after receiving instructions on how to use it, they performed teeth brushing with use of the application and digital device for a week, prior to a further set of PCR measurements being performed (C). The PCR score (A–B) of the control group in this study decreased by 9.4% from baseline, while in the intervention group, the score (A–C) decreased by 16%. Thus, assuming that the use of the brushing-instruction digital device results in a 6% decrease in the PCR score, with the level of significance (α) set at 5% (two-sided level) and the detection power (1–β) at 80%, the required participants’ number in one group was 63. Moreover, we estimated a drop-out rate of 10%, a power calculation indicated that a sample of 70 participants in each group was required to detect intergroup differences.

### Randomization

For the randomization process, the stratified block randomization method (block size; six) was used, where participants were randomly assigned to either of the two groups in a 1:1 ratio using stratification methods according to gender (male/female) and baseline PCR score (≥ 60%/< 60%) with a computer-generated randomization list, by a trial staff who only took charge of randomization. Randomization code was prepared in advance by the trial statistician. When an eligible participant was identified, the trial staff checked his/her teeth report and allocated using the randomization list.

### Blinding

Both the assignment process and the explanation of the digital devices were conducted in a protected area, and randomization concealment for the group was maintained for the dental hygienists who measured PCR score and for the analysts of the data. On the other hand, we could not blind for the participants.

### Statistical analysis

Participants characteristics were compared between the two groups using the *t*-test for continuous variables and the Chi-square test for categorical variables. In evaluating the primary and secondary outcome, changes in PCR score between baseline and week 4 were assessed using the *t*-test. In addition, we divided the whole tooth into three surfaces parts (medial and distal surfaces, buccal center surfaces and lingual center surfaces) and two teeth parts (anterior teeth and posterior teeth), and calculated the mean PCR score at baseline and week 4, and the presence of changes in each part. The differences in the consciousness of brushing and the time spent brushing between the two groups were evaluated using the Mann-Whitney *U* test.

We also calculated the number of participants with plaque on each tooth surface at baseline, and the number of participants who still had plaque after 4 weeks, as well as the decrease ratio, which was classified into 10% groups. Moreover, we calculated the number of the tooth surfaces of each group of the decrease ratio and the proportion of tooth surfaces relative to all 192 teeth surfaces.

All statistical analyses were performed on an intention-to-treat basis. All p values were two-sided, and p values < 0.05 were considered statistically significant. We used R statistical software (The R Foundation for Statistical Computing, ver. 3.5.2) [27].

## Results

**Fig 3:**
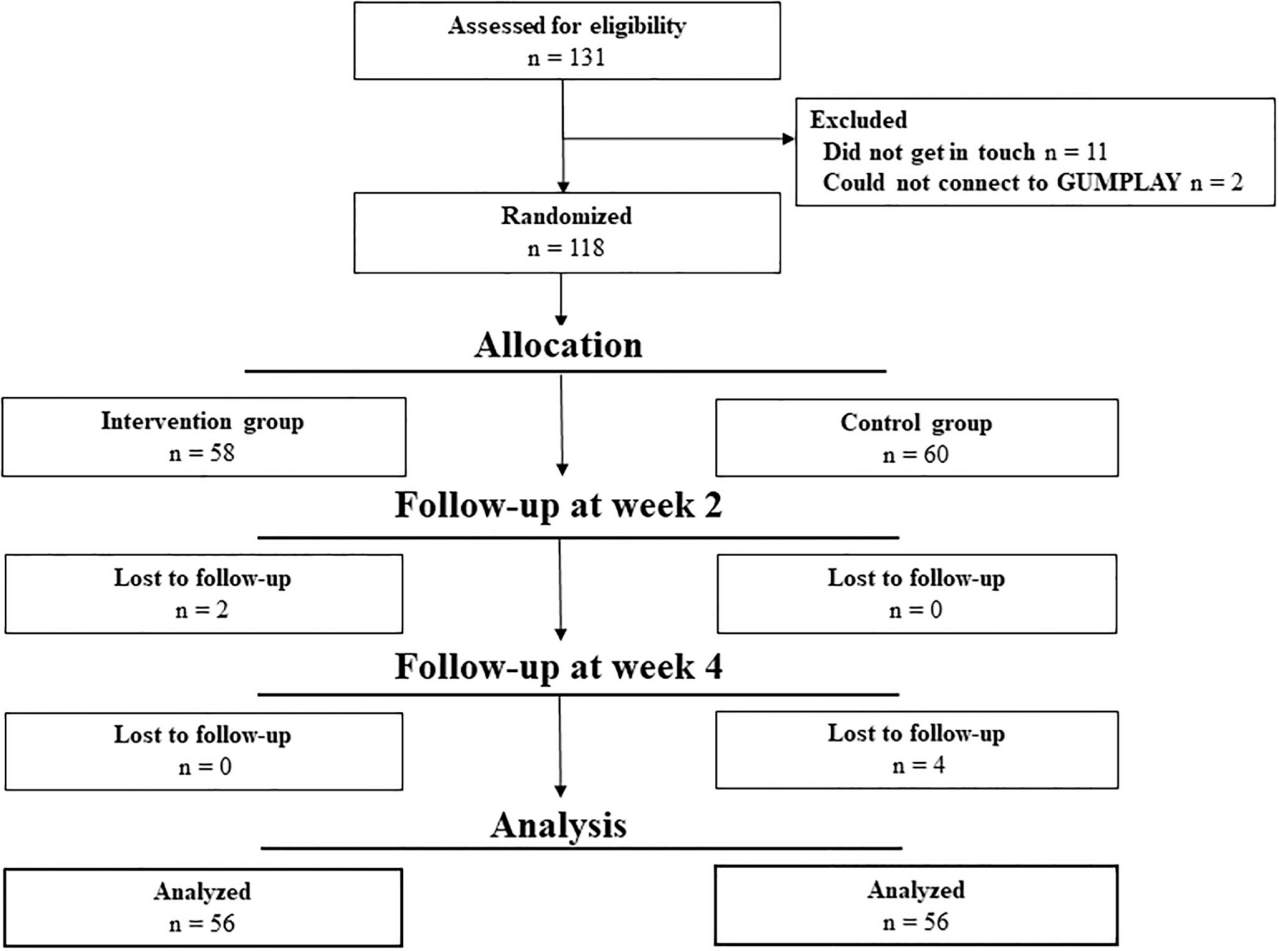
Participant flow in this trial.

The trial was conducted from October 29^th^, 2018 to November 30, 2018 (the last day of the follow-up for the participants). Fig 3 shows the participant flow in this trial. A total of 131 participants were initially registered to take part in this trial. After excluding 11 participants who did not get in touch and 2 participants who could not connect to the brushing-instruction digital device, the remaining 118 participants were randomly assigned to either the intervention or control group. Of the 118 participants, 6 dropped out during follow-up. A total 112 participants were finally eligible for our analyses and the number in each group was 56 participants.

Table 1 shows the characteristics of participants included in this trial. Most of the participants were college students and the mean age of participants was 26.0 years in the intervention group and 25.0 years in the control group. The proportion of participants with a baseline PCR score ≥60%, and the number of treated teeth, were not statistically different between the two groups.

**Table 1:** Participant characteristics.

	Control group		Intervention group		*p value*^a^
	(n = 56)		(n = 56)		
**Mean age, years (SD)**	25.0	(7.6)	26.0	(8.7)	0.511
**Male gender, n (%)**	29	(51.8)	27	(48.2)	0.850
**Occupation, n (%)**					0.306
**- Student**	36	(64.3)	29	(51.8)	
**- Graduate student/Research student**	12	(21.4)	17	(30.4)	
**- Staff of college**	8	(14.3)	8	(14.3)	
**- Others**	0	(0.0)	2	(3.6)	
**Baseline PCR > = 60%, n (%)**	33	(58.9)	33	(58.9)	1.000
**Number of treated teeth, mean (SD)**	3.0	(3.9)	3.4	(3.9)	0.611
**Examinable teeth surfaces**^b^**, mean (SD)**	171.0	(10.2)	170.0	(10.4)	0.622
**Participants with previously treated teeth, n (%)**	36	(64.3)	36	(64.3)	1.000

Abbreviations: PCR, plaque control record; SD, standard deviation.

^a^Comparisons between the 2 groups were evaluated with *t*-test for continuous variables and Chi-square test for categorical variables.

^b^The teeth surface of 6-point method

Table 2 shows the mean of the PCR score at baseline, week 2 and week 4 using 6-point and 4-point methods, and the results of the primary and secondary outcome measures. Regarding the primary outcome, the mean 6-point method PCR score at week 4 was 45.05% in the intervention group and 49.65% in the control group, and the change of mean PCR score from baseline to week 4 in each group was −20.46% in the intervention group and −15.77% in the control group, indicating no statistically significant difference (*p* = 0.088, 95% confidence interval [95%CI] −0.70–10.07). The change of mean PCR score from baseline using the 4-point method as the secondary outcome was significantly greater in the intervention group (−18.09%) compared to the control group (−13.11%; *p* = 0.047, 95%CI 0.07–9.90). The change of mean PCR score from baseline to week 2 was −13.53% in the intervention group and −10.26% in the control group. Moreover, the mean PCR score at week 4 decreased from baseline to a larger extent in the intervention group compared to the control group across all three surfaces parts (medial and distal surfaces, buccal center surfaces and lingual center surfaces) and two teeth locations (anterior teeth and posterior teeth; Table 3).

**Table 2:** Changes in PCR score between baseline and week 4.

		Control group		Intervention group		*p value*^a^	95%CI
**PCR (Full arch) [%], mean (SD): 6-point method**	BL	65.42	(18.85)	65.51	(18.07)		
	2W	55.16	(15.26)	51.98	(15.73)		
	4W	49.65	(17.47)	45.05	(14.98)		
	**Dif (4W–BL)**	**−15.77**	**(14.90)**	**−20.46**	**(13.86)**	**0.088**	**−0.70–10.07**
**PCR (Full arch) [%], mean (SD): 4-point method**	BL	69.34	(17.90)	69.47	(16.44)		
	2W	60.10	(14.50)	56.90	(14.52)		
	4W	56.24	(18.01)	51.38	(14.78)		
	**Dif (4W–BL)**	**−13.11**	**(13.20)**	**−18.09**	**(13.07)**	**0.047**	**0.07–9.90**

Abbreviations: 2W, week 2; 4W, week 4; BL, baseline; CI, confidence interval; Dif, difference; PCR, plaque control record; SD, standard deviation.

^a^Comparisons between the two groups were performed using a *t*-test analysis.

**Table 3:** Mean PCR scores at baseline and week 4 according to the tooth part and location.

Part/location	Group	Baseline		Week 4		Dif (4W–BL)	
**Part**							
**Medial and distal surfaces**	**Control**	73.32	(17.43)	56.92	(16.77)	−16.41	(16.18)
	**Intervention**	73.49	(17.37)	53.72	(16.41)	−19.76	(14.57)
**Buccal center surfaces**	**Control**	44.23	(26.24)	34.06	(24.87)	−10.18	(20.35)
	**Intervention**	47.19	(27.04)	29.17	(20.72)	−18.02	(24.25)
**Lingual center surfaces**	**Control**	54.94	(27.53)	36.14	(22.72)	−18.80	(19.20)
	**Intervention**	51.89	(25.72)	26.29	(17.05)	−25.60	(21.49)
**Location**							
**Anterior teeth**	**Control**	56.82	(23.36)	38.22	(21.26)	−18.60	(18.69)
	**Intervention**	57.24	(23.01)	32.49	(16.05)	−24.75	(17.97)
**Posterior teeth**	**Control**	71.65	(17.30)	57.86	(17.02)	−13.79	(14.66)
	**Intervention**	71.58	(16.54)	54.34	(16.52)	−17.25	(13.16)

Abbreviations: 4W, week 4; BL, baseline; Dif, difference; PCR, plaque control record.

Numbers in the table represent percentage (standard deviation).

A 91–100% decrease ratio in plaque from baseline to week 4 was observed in 5 tooth surface parts in the intervention group compared to none in the control group. The complete set of decrease ratios by group are shown in S1 Fig and S1 Table.

Table 4 shows the results of the questionnaire survey regarding the consciousness of brushing teeth and time spent brushing teeth. The distribution of each consciousness question (Q1-Q3) and brushing time were similar between the intervention and control groups at baseline. However, the number of participants who were consciousness of the brushing procedure after four weeks was 49 (87.5%) in the intervention group and 16 (28.6%) in the control group (*p*<0.001). Furthermore, the distribution of each consciousness and brushing time were different in the two groups at week 4. Some participants in the intervention group reported that, "I could learn the appropriate brushing methods." and that " The digital device gave me an opportunity to review my brushing" at week 4.

**Table 4:** Consciousness about brushing and brushing time.

	Baseline					Week 4				
	Control group		Intervention group		*p value*^a^	Control group		Intervention group		*p value*^a^
	(n = 55)		(n = 56)			(n = 56)		(n = 56)		
**Q1. Are you conscious of the brushing procedure?, n (%)**					0.806					<0.001
- Always consciousness	4	(7.3)	4	(7.1)		16	(28.6)	49	(87.5)	
- Sometimes consciousness	15	(27.3)	14	(25.0)		35	(62.5)	7	(12.5)	
- Not consciousness	36	(65.5)	38	(67.9)		5	(8.9)	0	(0.0)	
**Q2. Are you conscious of brushing each tooth surface?, n (%)**					0.384					<0.001
- Always consciousness	16	(29.1)	22	(39.3)		31	(55.4)	52	(92.9)	
- Sometimes consciousness	24	(43.6)	20	(35.7)		23	(41.1)	4	(7.1)	
- Not consciousness	15	(27.3)	14	(25.0)		2	(3.6)	0	(0.0)	
**Q3. Are you conscious of moving the toothbrush in small steps?, n (%)**					0.607					<0.001
- Always consciousness	14	(25.5)	15	(26.8)		19	(33.9)	42	(75.0)	
- Sometimes consciousness	22	(40.0)	25	(44.6)		34	(60.7)	14	(25.0)	
- Not consciousness	19	(34.5)	16	(28.6)		3	(5.4)	0	(0.0)	
**Q4. For how many minutes are you brushing each time?, n (%)**					0.476					<0.001
- 1 minute	10	(18.2)	11	(19.6)		0	(0.0)	0	(0.0)	
- 2 minutes	27	(49.1)	21	(37.5)		21	(37.5)	3	(5.4)	
- 3 minutes	18	(32.7)	24	(42.9)		35	(62.5)	53	(94.6)	

^a^Comparisons between the two groups were performed using the Mann–Whitney *U* test.

During this trial, adverse events and serious harms were not observed.

## Discussion

In this RCT, we evaluated the effects of the digital device, which offers real-time visualized brushing instructions. We found that the 6-point method PCR score decreased 4.69% more after four weeks by using the digital device with visualized brushing instructions compared to usual brushing instructions alone, although the difference was not statistically significant. The PCR score calculated using the 4-point method as the secondary outcome measure was 4.98% lower through the use of the digital device with visualized brushing instructions compared to usual brushing instructions alone, and this difference was statistically significant. In buccal center surfaces, lingual center surfaces and the anterior teeth, the PCR score at week 4 had a tendency to show a greater decrease in the intervention group compared to the control group. Our questionnaire survey data indicated behavioral change more in the intervention group participants compared to the control group participants. These results have important implications for the removal of dental plaque and habituation of appropriate brushing techniques.

Several previous studies have investigated the effect of plaque removal by brushing, providing instructions about brushing methods, and also the association between brushing time and plaque removal [8–11]. Schluter et al. investigated how to master the appropriate brushing methods by using leaflets or demonstrations, however their results showed the difficulties in mastering brushing methods [11]. Moreover, Greeth et al. showed that brushing time is an important determination of plaque removal [14]. In our trial, participants in the intervention group were able to take more time for brushing their teeth by using the digital device with instructions of the teeth parts where most people perform insufficient brushing. Therefore, mastering the appropriate brushing methods and enough brushing time may have influenced the PCR score decrease at week 4. The 4-point method PCR score used as the secondary outcome is measured commonly in regular clinical practice. It could be argued that the results of the PCR score change using the 4-point method were clinically significant. However, we used 6-point method PCR score as the primary outcome in order to evaluate plaque removal in more detail, including evaluation of the medial and distal surfaces. The smallest differences between the intervention and control groups in the PCR score were observed at the medial and distal surfaces compared to the other surfaces, and this might be one of the explanations why we could not find statistically differences in the primary outcome analysis. Based on this result, we should further investigate what instructions can be used to achieve plaque removal at the medial and distal surfaces, where it is known to be difficult to remove plaque.

We used the same video material for all participants receiving tooth brushing instructions in this trial, therefore we were unable to personalized instructions based on each participant’s oral condition. It may be more effective, even in difficult parts of the mouth, to learn an individual’s oral condition and provide suitable brushing methods under professional instruction.

Plaque control is recognized as an effective approach to prevent periodontal disease, and the prevention of periodontal disease can result in the prevention of many lifestyle-related diseases [3–6]. Recently, periodontal disease in young people has become commoner [1,2], and thus the role of early intervention with brushing instructions has become increasingly important. Use of the brushing-instruction device with the digitalized application may be a useful trigger for self-care in the generations that do not have as much awareness of teeth brushing.

The PCR score tended to decrease more from baseline in the intervention group compared to the control group. The decrease ratio of PCR score on each tooth surface part tended to be greater in the intervention group than the control group. However, in several parts most participants could not remove the plaque and it was difficult to achieve the appropriate PCR score regardless of the use of the brushing-instruction digital device. Guidelines recommend keeping the PCR score between 10% and 20% in order to prevent periodontal disease [1,26], however the average PCR score in our trial was over 20%. Matsunaga’s study showed that it is difficult to keep the PCR score under 10% and that, on average, individual instructions by professionals needed to be provided 3.8 times to achieve a PCR score under 10% [28]. Self-care is considered essential for plaque control [1], but alone it is insufficient and professional care is important.

In the questionnaire survey at week 4, most participants in the intervention group stated that they are “always conscious” of the procedure of teeth brushing, of brushing each surface of the tooth, and moving the toothbrush in small steps. This suggests that participants may have an increased awareness of appropriate brushing techniques due to instructions from the digital device. It is important to continue appropriate teeth brushing every day to enable plaque control, and motivation as well as repeated brushing instructions are required for this. As can be seen from our results of decreasing the mean of the PCR score at week 4 from week 2, the use of the digital device, which provides repeated real-time visualized brushing instructions, may be effective for people to get habituation of continuous brushing their teeth properly. However, it remains to be seen whether participants can maintain the consciousness of appropriate teeth brushing after this trial, and further investigations are needed to evaluate this.

This trial has several inherent limitations. First, the number of participants did not satisfy the sample size we calculated beforehand, because of the difficulties in recruitment. Second, masking participants was impossible in our trial because of the different brushing approaches in the intervention and control groups. Third, the Hawthorne effect may have influenced our trial results, with participants performing better with teeth brushing because of awareness that they are being monitored in a trial setting. Fourth, the follow-up period of this trial was restricted. It is assumed that people lose interest for the digital device as they use it for long time. Therefore, we cannot discuss whether the effect of this digital device continues, if people use it for a long time. Finally, the generalizability of our results is limited by the fact that this was a single-center trial with several inclusion criteria. This shows that we only investigate the restricted population, moreover, the most participants were university students and the mean of their age was 20s. Generally, the proportion of the people who have periodontal disease is said to be high in elderly persons compared to that of young persons. Therefore, the findings of this trial may not apply to elder population.

## Conclusion

We observed a non-significant reduction in the 6-point method PCR score after 4 weeks of using the digital device with visualized brushing instructions, when compared to usual brushing alone. However, the consciousness of teeth brushing at week 4 improved more in the intervention than control group. The use of a digital device providing real-time visualized brushing instructions may be somewhat effective for the removal of dental plaque and habituation of appropriate brushing, and may be useful as a trigger for self-care.

## Supporting information

S1 Fig The decrease ratio between baseline and week 4 on each tooth surface in the participants who had plaque. (XLSX)

S1 Table Number of tooth surfaces in each group of the decrease ratio between baseline and week 4 in participants who had plaque. (XLSX)

S1 Checklist CONSORT checklist. (DOC)

S1 Protocol (DOCX)

S2 Protocol (DOCX)
