# Supplementary material for: Effectiveness of a digital device providing real-time visualized tooth brushing instructions: A randomized controlled trial
Source: PLoS One. 2020 Jun 25;15(6):e0235194. doi: 10.1371/journal.pone.0235194 (PMC7316295; doi:10.1371/journal.pone.0235194)
Supplement: S1 Protocol — (DOCX) [file pone.0235194.s004.docx]

**Research Protocol**

**Effectiveness of a digital device providing real-time visualized tooth brushing instructions: A randomized controlled trial**

Ver.1 May 8^th^, 2018

Ver.2 July 5^th^, 2018

Ver.3 August 24, 2018

Principal investigator (study supervisor)

Taku Iwami^1^

Investigators

Haruka Shida^2^, Satoe Okabayashi^1^, Takashi Kawamura^1^

1. Kyoto University Health Service
2. Department of Preventive Services, Kyoto University School of Public Health
3. **Background**

Periodontal disease is an inflammatory condition caused by bacterial infection. Decayed teeth and periodontal disease represent the two major causes of tooth loss in dentistry. According to the National Survey of Dental Disease 2016 by the Ministry of Health, Labour and Welfare in Japan, the proportion of individuals with periodontal pockets ≥4 mm that develop into periodontal disease is 25.7% of young adults aged 20-24 years.  However, the proportion is 54.1% among those aged ≥50 years ^1^, and there has been a rapid rise in this proportion between 2011 and 2016 ^1,2^. The onset and progression of periodontal disease results in tooth loss, which triggers oral dysfunction that can adversely affect not only teeth and oral health but also systemic health ^2^. In particular, it has become apparent that periodontal disease is related to cardiovascular diseases such as coronary artery disease and stroke, as well as lifestyle-related diseases such as atherosclerosis and diabetes ^6^. Furthermore, many reports suggest that pneumonia, which is common in the elderly, occurs at a high rate in elderly people with poor oral hygiene ^7^. As such, the treatment and prevention of periodontal disease is an important societal issue.

Removal of plaques on the surface of teeth and plaque control are effective in the prevention and treatment of periodontal disease. Tooth care is broadly divided into self-care and professional care, the latter being performed by dental professionals ^2,8^. The simplest self-care that can be performed is mechanical plaque control, with the use of cleaning tools such as a toothbrush. The removal of plaque by tooth brushing and brushing instructions has been shown to be effective ^9^. The guidelines for periodontal disease treatment emphasize the importance of motivation, self-care, brushing instructions, and professional care in plaque control ^2^. Various interventions for plaque control have been developed. Brushing instructions are important for appropriate brushing techniques during self-care, and can be provided by the dentist or dental hygienist during dental examinations; however, few people undergo regular dental examinations and thus few people acquire the ideal brushing skills ^10^. The brushing method commonly used in the clinical setting for instructing patients in the prevention and treatment of periodontal disease is called the Bass method ^11^, which requires one to move the toothbrush in small steps. However, it has been pointed out that it is difficult to master this skill^11,12^. In previous research on the methods for learning brushing skills, few participants could master the appropriate brushing methods and skills ^12^. Furthermore, for plaque to be removed, it is preferable for one to perform brushing for a minimum of two minutes^13^. However, it has been reported that only about 20% of individuals actually satisfy the recommended brushing time^10^. In the modern busy lifestyle, it is difficult for one to get into the habit of brushing for a fixed amount of time.

Given the above, at Sunstar Inc. (Osaka, Japan), a device called GUMPLAY^®^ was developed to provide visible brushing instructions. This device is made up of a body that is linked to a mobile application. The device is installed in the bottom of a toothbrush, and once the mobile application is switched on, it not only provides brushing instructions through the built-in game element, it provides motivation for self-care; indeed, the device was originally developed for this purpose. Similar applications and devices have been previously developed, but few of these have shown effectiveness. There is no evidence that receiving brushing instructions visually, using the previously developed applications and devices for daily toothbrushing, is associated with behavioral changes or the prevention of periodontal disease. In this study, we perform an experiment using GUMPLAY^®^, with a focus on the acquisition of the appropriate brushing habits.

1. **Objective**

To investigate whether brushing of teeth using GUMPLAY^®^, a device that provides visualized brushing instructions through a linked mobile application, would contribute to the removal of dental plaque, and to the learning of appropriate brushing techniques over usual brushing.

1. **Method of selecting the study participants**

1） Setting

The study will be conducted at Kyoto University.

2）   Eligibility criteria

1. Inclusion criteria: Individuals aged 18 years and above.
2. Exclusion criteria: Individuals who met one or more of the following criteria were excluded from our study:

- Severely overcrowded teeth (one third or more of the teeth are overlapping, making it difficult to remove dental plaque using a toothbrush alone)
- Daily use of an interdental cleaning instrument
- An external injury or stomatitis that can affect teeth brushing
- Less than 20 remaining teeth
- Use of orthodontic apparatus
- Visit to a dental clinic within the past month
- Likely visit a dental clinic during the study period
- Possession of a dental license
- Non-ownership of a smart phone or tablet device
- A current smoker
- Use of oral antibiotics within the past week
- Potentially pregnant
- Allergy to the staining fluid used for dental plaques
- Employee of Sunstar Inc.

1. The scheduled number of participants and the rationale for the setting
2. Target number of participants: 95 in each group (190 in total)
3. Rationale for the calculation:

The target number of participants in this study was calculated based on the results of a pilot study conducted by Sunstar Inc. In the pilot study, the subjects were 9 adults aged ≥18 years old who were observed for two weeks. The plaque control record (PCR; the method for determining the plaque status in the tooth cervix) was measured (A) followed by brushing instructions from a dental hygienist. One week later, PCR was re-measured (B). On the same day, GUMPLAY^®^ was supplied to the subjects and, after receiving instructions on how to use it, they performed teeth brushing with use of the application and digital device for one week, prior to a further set of PCR measurements being performed (C).

The PCR score (A-B) of the control group in this study decreased by 9.4% from baseline, while in the intervention group, the score (A-C) decreased by 16%. Thus, assuming that the use of GUMPLAY^®^ results in a 6% decrease in the PCR score, with the level of significance (α) set at 5% (two-sided level) and the detection power (1－β）at 80％, the required number of required participants in one group is 63. Once a drop-out rate of 10 % is incorporated into the model, the number of required participants in each group is estimated to be 70. In addition, if the detection power (１－β) is set at 90%, the required number of participants in each group becomes 85 and, with a drop-out rate of 10%, the required number of subjects in each group is estimated to be 95. From the above, the target number of participants in both groups was set at 190, with a minimum of 140 becoming study subjects.

1. **Study methods and basis of the scientific ethical rationale of the study**
2. Design

Randomized controlled trial

1. Methods

- Registration procedure

The Steering Committee of this study will recruit participants from Kyoto University and provide an explanation of the study to each individual wishing to participate. After the explanation is complete, participants will be allowed to provide written informed consent and complete a pre-study questionnaire, which will be collected by the Steering Committee. The collected written informed consent forms and pre-study questionnaire will be collected, and the participants will be registered into the study.

1. Intervention methods

Prior to the start of the study, a dental hygienist will perform teeth surface cleaning to remove plaque in both groups of participants, and provide brushing instructions. Participants will be made to watch video teaching materials, to ensure homogeneity in the instructions given. After that, the intervention group will install a digital device (GUMPLAY^®^) linked with a mobile application onto the bottom of a toothbrush and perform teeth brushing under guidance of the application. This device: (1) enables visualization of the toothbrush position and provides a guide for the brushing sequence during teeth brushing, (2) ensures the brushing time lasts for at least 3 minutes, and (3) enables the user to visualize whether he or she is moving the toothbrush in small steps. The control group will install the digital device (GUMPLAY^®^), that functions only to collect brushing logs, to the bottom of a toothbrush and perform regular teeth brushing without using the mobile application. Both groups of participants will use a standard toothpaste (Ora^2^ me STAIN CLEAR, Sunstar Inc., Osaka, Japan) and toothbrush (GUM dental brush #166M, Sunstar Inc., Osaka, Japan).

1. Method of randomization

For the randomization process, the stratified permuted block randomization method will be used, where participants are stratified by gender (male / female) and baseline PCR score (≥60% / below 60%). Randomization codes in each stratum will be prepared in advance, and those participants that meet the study participation criteria based on the baseline measurement results shall be assigned to one of the two groups.

1. Blinding method

The participants will not be blinded (open-label), while randomization concealment for the group will be maintained for the dental hygienist who performs 3 oral examinations, and the data analyst.

1. Test items

- Baseline data
- PCR score
- Outcome data and measurement timing
- PCR score (week 2 and week 4)
- Teeth brushing speed and amplitude (the target is week 4/some participants: 10 participants in each group) during brushing.
- Patient background information

Age, gender, date of birth, profession, history of dental clinic visits, status of use of interdental cleaning instrument, and status of oral disease.

- Information on teeth brushing
- Frequency of teeth brushing in one day (obtained from GUMPLAY^®^)
- Teeth brushing time (obtained from GUMPLAY^®^)
- Daily teeth brushing record (obtained using a
- questionnaire)
- Questionnaire

*Initial questionnaire*

- Are you conscious of the brushing procedure?
- Are you conscious of brushing each tooth surface?
- Are you conscious of moving the toothbrush in small steps?
- For how many minutes are you brushing each time?
- Do you have the habit of doing something else while slowly brushing your teeth, and not focussing on just teeth brushing?
- How often do you normally brush your teeth in a day?
- Are you confident about the level of cleanliness inside your mouth?
- Are you confident about the way you brush your teeth?
- Do you feel that teeth brushing is bothersome?
- Do you presently have any concerns about the inside of your mouth?
- Are you currently interested in oral health?

*Questionnaire after the end of the study (intervention group)*

- In the past month, have you been conscious of the brushing procedure?
- In the past month, have you been conscious of brushing each tooth surface?
- In the past month, have you been conscious of moving the toothbrush in small steps?
- In the past month, for how many minutes have you been brushing each time?
- In the past month, how often have you been brushing your teeth regularly in a day?
- Compared to before the study, are you confident about the level of cleanliness of the inside of your mouth?
- Compared to before the study, are you confident about the way you brush your teeth?
- In the past month, have you felt that teeth brushing is bothersome?
- Do you presently have any concerns about the inside of your mouth?
- Are you currently interested in oral health?
- Do you now brush your teeth more properly than before, because you have used the GUMPLAY^®^ application?
- Has your motivation towards teeth brushing increased because you have used the GUMPLAY^®^ application compared to before using it?
- Did you feel that brushing (about 3 minutes) when using the GUMPLAY^®^ application was long?
- Would you like to continue using the GUMPLAY^®^ application hereafter also?
- Were you tired of using the GUMPLAY^®^ application?
- Would you recommend the GUMPLAY^®^ application to someone?
- What do you consider as good points and points of improvement of the GUMPLAY^®^ application?
- During the study period, did you visit a dental clinic?
- During the study period did you administer an antibiotic?

*Questionnaire after the end of the study (control group)*

- In the past month, have you been conscious of the brushing procedure?
- In the past month, have you been conscious of brushing each tooth surface?
- In the past month, have you been conscious of moving the toothbrush in small steps?
- In the past month, for how many minutes have you been brushing each time?
- In the past month, have you been in the habit of doing something else while slowly brushing your teeth, and not focussing on just the teeth brushing?
- In the past month, how often have you been brushing your teeth regularly in a day?
- Compared to before the study, are you confident about the level of cleanliness of the inside of your mouth?
- Compared to before the study, are you confident about the way you brush your teeth?
- In the past month, have you felt that teeth brushing is bothersome?
- Do you presently have any concerns about the inside of your mouth?
- Are you currently interested in oral health?
- During the study period, did you visit a dental clinic?
- During the study period did you administer an antibiotic?

1. Schedule


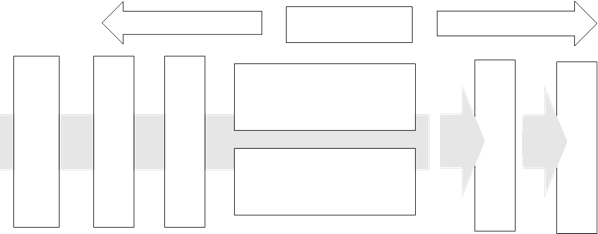


Group using the GUMPLAY^®^ linked to a mobile application

Study duration: 4 weeks

Second examination

Third examination

Group not using the GUMPLAY^®^ linked to a mobile application

Random assignment of participants

First examination

Enrollment to participate into the study

2 weeks

2 weeks

1. Summary of the analysis

- Primary outcome:
- PCR score at week 4 (using the 6-point method: evaluation of 6 parts of each tooth; the participants analyzed will be the Full Analysis Set)
- Secondary outcomes:
- PCR score at week 4 (using the 6-point method: evaluation of 6 parts of each tooth; the participants analyzed will be the Per Protocol Set)
- PCR score at week 2 (using the 6-point method: evaluation of 6 parts of each tooth)
- PCR score (using the 4-point method: evaluation of 4 parts of each tooth)
- Brushing time in one day
- Frequency of brushing in one day
- Method of evaluation: The PCR score will be evaluated by three dental hygienists using plaque staining fluid. The same dental hygienist will perform the baseline and outcome measurements of the same participant. In addition, to minimize inter-evaluator differences, the dental hygienists will be educated in advance, and calibration will be performed. Brushing speed and amplitude will be measured for some participants who give their consent to do so. For the measurements, brushing will be performed using a specialized toothbrush connected to a measuring device using a code.
- Method of analysis: The analysis will consist of two entities, a Full Analysis Set (FAS) and a Per Protocol Set (PPS).
- FAS

The analysis set of the primary and secondary outcomes. For the respective items, the mean value of the change from baseline in the intervention and control groups will be compared based on an intention-to-treat analysis. The estimated value of the difference between the 2 groups (intervention group－control group) and the 95% confidence intervals will be calculated. The p-values of the t-test will calculate the null hypothesis that the true intergroup difference is not different from zero. The significance level of the test will be 5%. A stratified analysis will be performed using the patients’ background information (e.g. frequency of brushing in one day).

- PPS

The analysis set of the secondary outcome. An individual to whom one or more of the following conditions apply will be excluded from the FAS:

1. A participant who visited a dental clinic during the study period.
2. A participant who administered an antibiotic during the study period.
3. A participant in the intervention group whose 4-week history of using GUMPLAY® did not show the at least once daily usage for more than 5 days　(GUMPLAY® was not switched on).
4. A participant in the control group whose 4-week history of using GUMPLAY® (one that functions only to collect brushing log) did not show the at least once daily usage for more than 5 days　(GUMPLAY® was not switched on).

The same analysis performed for the FAS will be performed for the PPS.

1. **Study period**
2. Participant enrollment period: Twelve months after obtaining the approval of the ethics committee, until the required number of participants is reached.
3. Participant observation period: Thirteen months (4 weeks after enrollment into the study) after obtaining the approval of the ethics committee
4. Study period: Thirty-six months from the date approval is received from the ethics committee.
5. **Informed consent**
6. Acquisition of new samples and information to conduct the study

In this study, a non-invasive intervention will be performed and, to conduct the study using newly acquired samples and information, the informed consent of the participants will be obtained after providing them with a written explanation.

The Investigator will provide a written explanation of the study to the participants and after they adequately understand the content, will obtain their informed consent based on their free-will prior to participating in the study. Prior to acquiring the informed consent, the participants will be given adequate time and an opportunity to ask questions, which will be answered adequately, for them to decide whether or not to participate in the study. The Investigator who provides the explanation and the participant will enter their name, signature and date in the informed consent form. The informed consent will be retained at the trial site and a copy of the written explanation and informed consent form will be handed to the participant.

1. Re-acquisition of informed consent if the protocol is amended

When an amended of the protocol etc. is made that will affect the consent of the participants, the information shall be promptly provided to the participants and their willingness to continue participating in the study confirmed in advance. In addition, the approval of the trial site director shall be obtained based on the opinion of the trial site ethics committee in advance. The written explanation and informed consent forms should then be revised and the informed consent of the participants obtained again.

1. **Handling of personal information**
2. Timing and methods for anonymizing samples

Participants who gave their informed consent to participate in the study will be assigned a personal study ID in the order of their enrollment. A Correspondence Table will then be prepared. The task shall be assigned to a dedicated manager and no other person will be allowed to come into direct contact with the personal information.

1. Management of the Correspondence Table

The Correspondence Table shall be stored securely in a locked box in a locked room by the designated manager of the Kyoto University Health Service Center.

1. Handling of data after a consent is withdrawn

In the event that a participant wishes to withdraw his or her consent after being enrolled in the study, the designated manager of personal information shall link the participant’s personal information with study ID and erase all the data of the relevant participant.

1. Items of personal information, safety control measures and points of note used or retained in a joint study

Data obtained from GUMPLAY^®^ shall be stored in an independent server by the same company entrusted by Sunstar Inc. The designated manager of Kyoto University will then download the data using an ID and password. After downloading the data, the data on the server will be irrecoverably deleted. Until the data is deleted, the Sunstar manager may inspect the data during the maintenance inspection and data management process. However, he or she cannot manipulate the data and will not use the data.

Moreover, only the results obtained after the analysis will be submitted to Sunstar Inc., which is the joint trial facility.

1. **Burden on the participants as well as the risks and benefits**
2. Burden and risks

Using GUMPLAY^®^ to perform teeth brushing on a daily basis will give rise to a burden in terms of time.

While using GUMPLAY^®^, the application is installed and using it gives rise to internet connection costs.

1. Benefits

The habit of brushing is encouraged, and that is linked to possible prevention of conventional dental diseases.

1. Overall evaluation of burden/risk and benefits, and measures to minimize the burdens and risks

Prior to enrollment into the study, the study content, survey content, time required, that are documented in writing and in publicly disclosed information shall be explained and the participants allowed to consider of their own free will whether they wish to participate in the study. In addition, the participants will be fully informed so that opportunities for them to provide written consent to participate in the study, as well as to withdraw written consent, will be given. Moreover, with regard to monetary burden, the participants will be given an honorarium and supplied with the GUMPLAY^®^ device after the study is completed.

1. **Methods to retain and dispose of samples and information**
2. Sample/information retention period

Samples/information obtained in the study shall be retained for at least 10 years from the day the final study results are reported.

1. Method for retaining samples/information

Samples and information obtained in the study will be retained in a keyed desk (can be locked using an electronic key) in the research room of the Kyoto University Health Service Center and managed such that no leakage, mixing, theft, loss etc., occurs.

1. The process when they are to be disposed after the end of the study

After 10 years have passed, data on printed medium shall be disposed of using a shredder, and data on an electronic medium shall be put into an unrecoverable state.

1. **Secondary use of samples and information and the possibility of submitting them to another research facility**

None.

1. **Reporting to the director of the trial site and the procedure**
2. In the event of loss of the ethical validity and scientific rationality of the study, or information that will likely get lost is obtained, a safety information report on the fact shall be promptly performed.
3. In the event that the propriety of the conduct of the study and reliability of the study results are lost or information that may be lost is obtained, a nonconformity report shall be promptly submitted.
4. Annual reporting shall be performed once every 3 or more years. Discontinuation or completion of the reporting shall be performed appropriately.
5. **Funding of the study and conflict of interest**
6. Type of study funding and the sponsor

Grant-in-aid for a joint study (sponsor: Sunstar Inc.)

1. Relationship of the sponsor and researchers

Co-investigator

1. Conflict of interest

This study will be conducted using the grant-in-aid for a joint study by Sunstar Inc. as the source of funding. There is no conflict of interest between the investigators and Sunstar Inc., the manufacturer of GUMPLAY^®^, the study device in this project. GUMPLAY^®^ was supplied and used to conduct the study, however, there is no other conflict of interest to declare. As there is the possibility of a conflict of interesting arising regarding the conduct and results of the study, based on the conflict of interest management policy of Kyoto University, this study shall be reviewed and approved by the conflict of interest review committee prior to the conduct of the study.

1. **Responding to consultations from participants and other related individuals**
2. Study contact person

Preventive Services, Kyoto University School of Public Health

Investigator: Haruka Shida

(E-mail) hamigaki_rct2018@hc2.hoken.kyoto-u.ac.jp

1. Kyoto University consultation contact person

Research Promotion Division, General Affairs and Planning Section, Kyoto University

(Tel) 075-753-9301　 (E-mail) kikaku06@mail2.adm.kyoto-u.ac.jp

Agency for Health, Safety and Environment, Kyoto University

(E-mail) 810kikochosei@mail2.adm.kyoto-u.ac.jp

1. **Economic burden and honorarium of the participants**

- A participant who completes all measurements in weeks 2 and 4 will be given a 5000 Yen QUO card (about 1,000 Yen for each hour of participation) as honorarium. (To some of the participant for whom speed and amplitude were measured will be given an 6000 Yen QUO card)
- At the end of the study each participant will be given a GUMPLAY^®^.

1. **Handling of study results (including incidental findings) related to the participants**

During the oral examination of the participants in this study, incidental findings such as inflammation may occur. In the event that a visit to a dental clinic is recommended, the dental hygienist shall inform the participant of that fact. There is no possibility that important findings related to genetic characteristics can be obtained.

1. **Study conduction system**
2. Principal investigator (study supervisor)

Prof. Taku Iwami: Kyoto University Health Service

Yoshida Honmachi Sakyo-ku, Kyoto, Japan

(Tel) 075-753-2426

(E-mail) iwami.taku.8w@kyoto-u.ac.jp

1. Investigators
2. Haruka Shida (Protocol making, management, analysis, paper writing)

Kyoto University School of Public Health

Yoshida Konoe cho Sakyo-ku, Kyoto, Japan

1. Satoe Okabayashi (Protocol making, management, analysis, paper writing)

Kyoto University Health Service, Assistant Prof.

Yoshida Honmachi Sakyo-ku, Kyoto, Japan

1. Takashi Kawamura (Advising for protocol making and paper writing)

Kyoto University Health Service, Prof.

Yoshida Honmachi Sakyo-ku, Kyoto, Japan

1. Study collaborators
2. Masami Yoshioka (Advising for protocol making)

Department of Oral Health Sciences, Faculty of Health and Welfare, Tokushima Bunri University, Prof.

1. Kosuke Kiyohara (Analysis)

Department of Food Science, Otsuma Women’s University, Lecturer

1. Daisuke Kobayashi (Allocation)

Kyoto University Health Service, Assistant Prof.

1. Tetsuhisa Kitamura (Effective and safety evaluation)

Department of Social and Environmental Medicine, Osaka University, Assistant Prof

1. Tasuku Matsuyama (Effective and Safety Evaluation)

Department of Emergency Medicine, Kyoto Prefectural University of Medicine, Assistant Prof

1. Chika Nishiyama (Effective and Safety Evaluation)

Department of Critical Care Nursing, Kyoto University, Lecturer

1. Co-investigator
2. Naoko Takase (Protocol making, management)

R&D Department, Sunstar Inc.

1. Yui Okazawa (Protocol making, management)

R&D Department, Sunstar Inc.

1. Masahiro Nishiura (Protocol making, management)

R&D Department, Sunstar Inc.

1. Shinji Matsutomi (Protocol making, management)

R&D Department, Sunstar Inc.

1. Name of Collaborating Institute

R&D Department, Sunstar Inc., Tokushima Bunri University, Otsuma Women’s University

1. **Research work outsourcing**

None

1. **Amendment and revision of the protocol**

In the event that an unexpected situation occurs, the protocol may need to be amended. If that occurs, the members conducting the study (Investigators) shall re-apply for the approval of the revised/amended protocol to the ethics committee of the Graduate School of Medicine and Faculty of Medicine Kyoto University Hospital, after appropriate deliberations.

1. **Ethical guidelines that must be complied to**

The investigators shall conduct the study in compliance with the World Medical Association Declaration of Helsinki, and respect the confidentiality of the participants and human rights in accordance with the Personal Information Protection Law and ‘Ethical Principles for Medical Research Involving Human Subjects’.

1. **Attribution and disclosure of the study results**

The study results belong to Kyoto University. The principal investigator is responsible for presenting the study results at academic meetings and magazines, as well as publishing them in peer-reviewed journals.

1. **Method of disclosing study-related information**

Pre-registration will be performed in the UMIN clinical study registration system of the University Hospital Medical Information Network (UMIN; Study ID：UMIN000034503).

1. **References**
2. Ministry of Health, Labour and Welfare. Survey of dental diseases. Available from: https://www.mhlw.go.jp/toukei/list/62-28.html.
3. Japanese Society of Periodontology. JSP Clinical practice guideline for the periodontal treatment, 2015. Tokyo: Ishiyaku Publishers, Inc.; 2016.
4. Khader YS．Periodontal diseases and the risk of coronary heart and cerebrovascular diseases: a meta-analysis. J Periodontol. 2004;75(8):1046-53.
5. Beck J, Garcia R, Heiss G, Vokonas PS Offenbacher S. Periodontal disease and cardiovasculardisease. J. Periodontol. 1996;67:1123-1137.
6. Grau AJ, Becher H, Ziegler CM, Lichy C, Buggle F, Kaiser C, Lutz R, Bultmann S, Preusch M Dorfer CE. Periodontal disease as a risk factor for ischemic stroke. Stroke. 2004;35:496-501.
7. 8020 Promotion Foundation. 歯周病と生活習慣病の関係, 2005. Tokyo: 8020 Promotion Foundation; 2005.
8. Pace C. C, McCullough G.H. The association between oral microorganisms and aspiration pneumonia in the institutionalized elderly: review and recommendations. Dysphagia. 2010; 25:307-322.
9. Crocombe LA, Brennan DS, Slade GD, Loc DO. Is self interdental cleaning associated with dental plaque levels, dental calculus, gingivitis and periodontal disease? J Periodont Res. 2012;47:188–197.
10. Poyato-Ferrera M1, Segura-Egea JJ, Bullón-Fernández P. Comparison of modified Bass technique with normal toothbrushing practices for efficacy in supragingival plaque removal. Int J Dent Hyg. 2003;1(2):110-114.
11. Ganss C1, Schlueter N, Preiss S, Klimek J. Tooth brushing habits in uninstructed adults--frequency, technique, duration and force. Clin Oral Investig. 2009;13(2):203-208.
12. Wainwright J, Sheiham A. An analysis of methods of toothbrushing recommended by dental associations, toothpaste and toothbrush companies and in dental texts. Br Dent J. 2014;217(3):E5.
13. Schlueter N, Klimek J, Saleschke G, Ganss C. Adoption of a toothbrushing technique: a controlled, randomised clinical trial. Clin Oral Investig. 2010;14(1):99-106.
14. Creeth JE, Gallagher A, Sowinski J, Bowman J, Barrett K, Lowe S, Patel K, Bosma ML. The Effect of Brushing Time and Dentifrice on Dental Plaque Removal in vivo. J Dent Hyg. 2009;83(3):111-116.
