# Supplementary material for: Effectiveness of a digital device providing real-time visualized tooth brushing instructions: A randomized controlled trial
Source: PLoS One. 2020 Jun 25;15(6):e0235194. doi: 10.1371/journal.pone.0235194 (PMC7316295; doi:10.1371/journal.pone.0235194)
Supplement: S2 Protocol — (DOCX) [file pone.0235194.s005.docx]

【研究課題名】

可視化したブラッシング指導を提供する　　　　　　デジタルデバイスを用いた無作為化介入試験

第1版　2018年5月8日

第2版　2018年7月5日

第3版　2018年8月24日

研究責任者：石見　拓^1^

研究実施者：志田　瑶^2^　、岡林　里枝^1^、川村　孝^1^

１）京都大学　環境安全保健機構 附属健康科学センター

２）京都大学大学院医学研究科　社会健康医学系専攻　予防医療学分野

1. 背景

歯周病は、細菌の感染によって引き起こされる炎症性疾患で齲蝕と並んで歯科の2 大疾患とされる。厚生労働省の平成28年歯科疾患実態調査によると、歯周疾患の目安となる歯周ポケットが4mm以上存在している割合が、20-24歳の若い年代でも25.7％、50歳以上では54.1％と半数を超える割合に達していた^1^。さらに年次推移を見ると、2011年から2016年の5年で急激に増加している^1,2^。歯周病の発症と進行によって歯の喪失等が生じると，口腔機能障害を引き起こし，歯や口腔の健康のみならず，全身の健康にも悪影響を及ぼすことが指摘されている^2^。特に冠動脈疾患や脳卒中などの心血管疾患^3-5^ 、動脈硬化や糖尿病などの生活習慣病^6^といった疾患との関連が明らかにされている。また高齢者に多い肺炎は、口腔衛生状態が不良な高齢者において発症する傾向が高いことが数多く報告^7^されており、歯周病の治療および予防への取り組みは社会における重要な課題となっている。

歯周病の予防・治療においては、歯の表面のプラーク（歯垢）を除去する、プラークコントロールが有効であるとされており、自分自身が行うセルフケアと歯科医療従事者が行うプロフェッショナルケアに大別される^2,8^。その中でも、もっとも簡単に行えるセルフケアは、歯ブラシなどの清掃用具による機械的なプラークコントロールであり、実際にブラッシングやブラッシング指導によるプラーク除去効果が示されている^9^。さらに歯周病治療のガイドライン上では、プラークコントロールにおける①モチベーション（動機づけ）、②セルフケア、③ブラッシング指導、④プロフェッショナルケアの重要性を指摘しており^2^、プラークコントロールに対する多角的な介入が求められている。そして、セルフケアにおいて理想的なブラッシングを実施するためには、ブラッシング指導が重要になると考えられる。ブラッシング指導は、歯科受診を行った際に歯科医師や歯科衛生士より指導を受けることができるが、定期的に歯科受診を行う人は少なく、理想とされるブラッシングを習得している人は少ない^10^。また、歯周病予防や治療の現場で実際に指導の際によく用いられているブラッシング方法はバス法と呼ばれ^11^、歯ブラシを小刻みに振動させる術式であるが、習得が難しいことが指摘されている^11,12^。先行研究において、ブラッシング方法の習得方法に関する研究が実施されているが、適切なブラッシング技術と手順を習得できたものは少ない^12^。さらに、プラークを除去するためには、ブラッシング時間は最低でも2分以上が望ましいとされている^13^。しかしながら実際に推奨されるブラッシング時間を満たす者は20%程度との報告もあり^10^、忙しい現代において、一定時間のブラッシングを習慣化することは困難であると考えられる。

このような背景を踏まえ、サンスター株式会社では、可視化したブラッシング指導を提供するデバイス（GUM PLAY）の開発を行った。本デバイスは本体と携帯アプリケーションから成り、本体を歯ブラシに装着し、携帯アプリケーションと連動させることにより、ブラッシングの指導を行うだけではなく、ゲーム要素等を加えることで、セルフケアへの動機づけを行うことを目的として開発された。今までにも類似するアプリケーションやデバイスの開発がおこなわれているが、その効果を示したものは少なく、日常のブラッシングにそれらのアプリケーションやデバイスを用いて視覚的にブラッシング指導を受けることが、行動変容や歯周疾患の予防に関連しているかはわかっていない。今回の試験では、適切なブラッシングの習慣化に焦点をあて、ゲーム要素を含まず、ブラッシング指導を行うことに特化したアプリケーションと連動するデバイスを使用し、試験を実施する。

1. 目的

携帯アプリケーションと連動させてブラッシング指導を行うことができるデバイス（GUM PLAY）を用いたブラッシングの方が、通常のブラッシングより、適切なブラッシングの習慣化や、プラーク除去に寄与するかを検証する。

1. 研究対象者の選定方法
2. セッティング

京都大学において実施する

1. 適格基準
2. 選択基準：18歳以上の者
3. 除外基準：以下のいずれかに該当する者を除外する

- 重度の叢生を認める者（歯と歯が3分の１以上重なり合っており、歯ブラシのみでのプラークコントロールが難しい者）
- 毎日歯間清掃具を使用している者
- ブラッシングに影響を与えるほどの外傷や口内炎などが認められる者
- 残存歯数が20本未満の者
- 口腔内に矯正装置が装着されている者
- 1カ月以内に歯科医院を受診した者
- 試験期間中に歯科医院を受診する可能性のある者
- 歯科系資格取得者
- スマートフォンもしくはタブレットを所持していない者
- 喫煙者
- 1週間以内に抗菌剤を内服した者
- 妊娠の可能性のある者
- 歯垢染色液成分に対するアレルギーを有する者
- サンスター株式会社の従業員である者

1. 予定研究対象者数及びその算定根拠
2. 目標症例数：片群95人（両群190人）
3. 算定根拠

サンスター株式会社によって、本試験の予定対象者数算定のために行われた予備調査の結果をもとに算出した。

予備調査では、18歳以上の成人9名を対象とし、2週間の観察が行われた。9名に対し、ベースラインとなるプラークコントロールレコード（歯頚部のプラークの有無を判定する方法。以下PCR）を測定し（A）、その後歯科衛生士によるブラッシングの指導を受け、1週間後のPCRを測定した（B）。同日にGUM PLAYを提供し、使い方を指導した後、1週間アプリと連動させたデバイスを用いてブラッシングを実施してもらい、PCRの測定を行った（C）。

その結果、本研究における対照群にあたるPCRの結果（A－B）は、ベースラインより9.4%の減少を認め、介入群にあたるPCRの結果（A－C）は16％の減少を認めた。よって、GUM PLAY使用により、6 %のPCRの減少を仮定し、有意水準（α）を5%（両側水準）、検出力（１－β）を80％とした場合、必要症例数は片群63名、脱落症例を10％と見積もると片群70名、また検出力（１－β）を90%とした場合、必要症例数は片群85人、脱落症例を10%と見積もると片群95名となる。以上から、目標症例数を両群190名、最低140名を研究対象者とすることとした。

1. 研究の方法及び研究の科学的合理性の根拠
2. デザイン

無作為化比較対照試験

1. 方法
2. 登録の手順

本研究運営メンバーが、京都大学で対象者を募集し、参加希望者に個別に説明する。この説明終了後に、研究同意書及び事前質問用紙に記入してもらい、説明を行った研究運営委員会メンバーが回収する。回収した研究同意書及び事前質問用紙を研究事務局に集め、登録を行う。

1. 介入方法

試験開始前に、両群に対して歯科衛生士がプラーク除去のための歯面清掃を実施し、更にブラッシング指導を行う（均質な指導となるようビデオ教材の視聴とする）。その後、介入群は、ブラッシングのガイドアプリ機能が付いたデジタルデバイス（GUM PLAY）の本体を歯ブラシに装着し、アプリのガイド下でブラッシングを行う。このデバイスは、ブラッシング時に①歯ブラシの位置の可視化およびガイド、②ブラッシング時間が3分間以上となるようガイド、③小刻みに歯ブラシを動かせているかを可視化、する機能を有する。対照群は、ブラッシングのログ収集機能のみを持つデジタルデバイス（GUM PLAY）の本体を歯ブラシに装着するのみで、ガイドアプリは使用せずに、通常どおりブラッシングを行う。両群とも試験期間中は指定のハミガキ（オーラツーミー ステインクリアペースト）、歯ブラシ（ガム・デンタルブラシ#166M）を使用する。

1. 無作為化の方法

割付け方法には、性別（男・女）・ベースラインPCR（60%以上・60％未満）で層別した層別置換ブロック法を用いる。予め各層にランダム化のコードを準備しておき、ベースラインの測定結果により、研究参加基準を満たした対象者を２群に割付ける。

1. マスキング方法

研究対象者は非マスク化（オープンラベル）、計3回の口腔診査を実施する歯科衛生士と解析担当者は、対象者がどちらの群に割り付けられているかわからないようマスク化を行う。

1. 調査項目
2. ベースラインデータ

- PCR

1. 転帰データと測定時期

- PCR（2週目・4週目）
- ブラッシング時の歯ブラシの加速度、振幅（4週目/一部の対象者：各群10名を目標とする）

1. 患者背景

年齢、性別、生年月日、職業、歯科医院の通院歴、歯間清掃具の使用有無、口腔疾患の有無

1. ブラッシングに関する情報

- 1日のブラッシング回数（GUM PLAYより取得）
- 1回のブラッシング時間（GUM PLAYより取得）
- 日々のブラッシングの記録（自記式質問紙を用いて取得）

1. アンケート

〇初回アンケート

- 歯みがきの時に磨く順番を意識しているか
- 歯の表面、裏面、かみ合わせ面のすべてを意識して磨いているか
- 歯ブラシを小刻みに動かして磨いているか
- 通常の歯みがき時間はどれくらいか
- 歯みがきだけに集中せず、何かをしながらゆっくりと歯をみがく習慣が　　あるか
- 通常の１日の歯みがき回数はどれくらいか
- 自分の口の中の清潔度に自信があるか
- 自分の歯みがきに自信があるか
- 歯みがきを面倒だと感じるか
- 現在、口の中に気になることはあるか
- 現在、口の中の健康に興味があるか

〇試験終了後アンケート（介入群）

- １か月間、歯みがきの時に磨く順番を意識していたか
- １か月間、歯の表面、裏面、かみ合わせ面のすべてを意識して磨いていたか
- １か月間、歯ブラシを小刻みに動かして磨いていたか
- １か月間、通常の歯みがき時間はどれくらいであったか
- １か月間、通常の１日の歯みがき回数はどれくらいであったか
- 試験前と比べて、自分の口の中の清潔度に自信があるか
- 試験前と比べて、自分の歯みがきに自信があるか
- １か月間、歯みがきを面倒だと感じることがあったか
- 現在、口の中に気になることはあるか
- 現在、口の中の健康に興味があるか
- GUM PLAYアプリ使用によって以前より丁寧に磨くようになったか
- GUM PLAYアプリを使用することで、使用前と比べて歯みがきへの意欲は高まったか
- GUM PLAYアプリ使用時の歯みがき（約3分程度）は長く感じたか
- GUM PLAYアプリを今後も使用し続けたいと思うか
- GUM PLAYアプリの使用に飽きたか
- GUM PLAYアプリを人に勧めたいと思うか
- GUM PLAYアプリについて良かった点や改善点について
- 試験期間中に歯科医院を受診したか
- 試験期間中に抗菌剤を内服したか

〇試験終了後アンケート（対照群）

- １か月間、歯みがきの時に磨く順番を意識していたか
- １か月間、歯の表面、裏面、かみ合わせ面のすべてを意識して磨いていたか
- １か月間、歯ブラシを小刻みに動かして磨いていたか
- １か月間、通常の歯みがき時間はどれくらいであったか
- １か月間、歯みがきだけに集中せず、何かをしながらゆっくりと歯をみがくことがあったか
- １か月間、通常の１日の歯みがき回数はどれくらいであったか
- 試験前と比べて、自分の口の中の清潔度に自信があるか
- 試験前と比べて、自分の歯みがきに自信があるか
- １か月間、歯みがきを面倒だと感じることがあったか
- 現在、口の中に気になることはあるか
- 現在、口の中の健康に興味があるか
- 試験期間中に歯科医院を受診したか
- 試験期間中に抗菌剤を内服したか

1. スケジュール


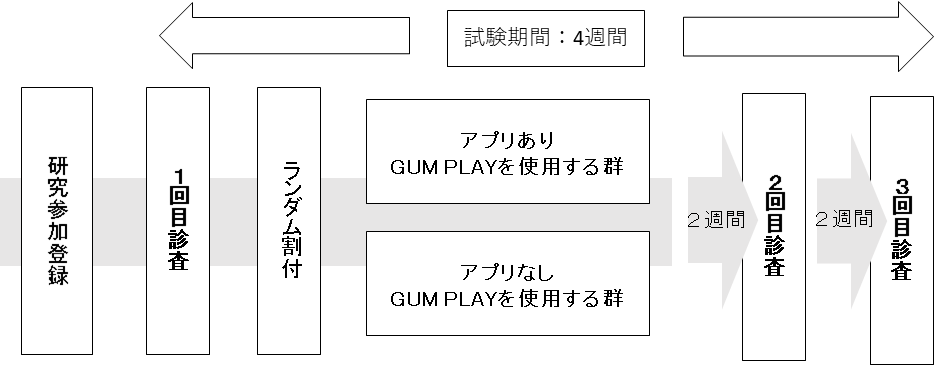


1. 解析の概要
2. 主要評価項目：

4週目のPCR（6点法：1歯につき6部位を評価）（解析対象をFull Analysis Setとする）

1. 副次評価項目：

・4週目のPCR（6点法：1歯につき6部位を評価）（解析対象をPer Protocol Setとする）

・2週目のPCR（6点法：1歯につき6部位を評価）

・PCR（4点法：1歯につき4部位を評価）

・1日のブラッシング時間

・1日のブラッシング回数

1. 評価方法：PCRに関しては、歯科衛生士3名が歯垢染色液を用いて評価を行う。その際に、各対象者のベースラインと転帰測定を行う歯科衛生士が同一人物になるようにする。また、評価者間の違いを小さくするために、評価する歯科衛生士に事前教育を行い、キャリブレーションを行う。加速度、振幅に関しては、同意を得た一部の対象者に対して計測する。計測にあたっては、計測装置にコードで接続された状態の専用歯ブラシを使用してブラッシングを行う。
2. 解析方法： 解析対象をFull Analysis Set（FAS）、Per Protocol Set（PPS）の２つ定めることとする。

〇FAS

主要評価項目および副次的評価項目の解析対象集団とする。Intention to treat解析に基づき、それぞれの項目に対してベースラインからの変化量について、介入群と対照群の平均値を比較する。2群間の差（介入群－対照群）の推定値とその95%信頼区間を計算し、群間差の真値が0であるという帰無仮説に対するt検定のp値を計算する。検定の有意水準は5%とする。患者背景情報（1日のブラッシング回数など）を用いて、層別解析を行う。

〇PPS

副次的評価項目の解析対象集団とする。FASから以下の条件に1つでも該当する者を除外する。

1. 試験期間中に歯科医院を受診した者
2. 試験期間中に抗菌剤を内服した者
3. 介入群の試験4週目のGUM PLAY使用履歴において、1日1回以上の使用が5日以上ない（GUM PLAYが起動されなかった）者
4. 対照群の試験4週目のGUM PLAY（ブラッシングのログ収集機能のみを持つもの）使用履歴において、1日1回以上の使用が5日以上ない（GUM PLAYが起動されなかった）者

解析方法はFASと同様に行うこととする。

1. 研究期間
2. 研究対象者登録期間：倫理審査承認後、1年間（必要対象者数に到達するまで）
3. 研究対象者観察期間：倫理審査承認後、1年1ヵ月間（研究登録後、4週間）
4. 研究実施期間：倫理審査承認日から3年間
5. インフォームドコンセント
6. 新たに試料・情報を取得して研究を実施する場合

本研究は、侵襲の伴わない介入を行い、かつ新たに試料・情報を取得して研究を実施するため、説明同意文書にて研究対象者への同意取得を行う。

研究担当者は、研究対象者が研究に参加する前に、説明文書を用いて十分説明し理解を得た上で、研究参加について自由意思による同意を研究対象者本人より文書で得る。同意を得る際には、研究対象者に研究に参加するか否かを判断するのに十分な時間と質問する機会を設け、質問に十分に答える。説明した研究担当者ならびに研究対象者は、同意書に記名・捺印または署名し、各自日付を記入する。同意書は実施研究機関で保存し、説明文書および同意書の写しは研究対象者に渡す。

1. 研究計画書を変更した際の再同意

研究対象者の同意に影響を及ぼすような研究計画等の変更が行われるときは、速やかに研究対象者に情報提供し、研究に継続して参加するか否かについて研究対象者の意思を予め確認するとともに、事前に研究機関の倫理審査委員会の意見に基づき研究機関の長の許可を得て説明文書・同意書の改訂を行い、研究対象者の再同意を得る。

1. 個人情報の取り扱い
2. 試料等の匿名化の時期と方法

研究参加への同意が得られた者には、登録順に個別の研究用IDを付与し、対応表の作成を行う。作業は、専任の担当者を置き、それ以外の者が個人情報に直接触れることはない。

1. 対応表の管理方法

対応表は、専任の担当者の元、京都大学環境安全保健機構健康科学センターの鍵のかかる部屋の鍵のかかる棚の中に、厳重に保管する。

1. 同意撤回後のデータの取り扱いについて

登録後に同意撤回の申し出のあった場合には、個人情報の専任担当者により研究用IDとの紐づけを行い、当該対象者のデータをすべて消去する。

1. 共同研究において保有または利用する個人情報等の項目と安全管理措置及び留意事項

GUM PLAYから取得されるデータは、サンスター株式会社が委託した同社から独立したサーバーに保存し、京都大学の専任の担当者がIDとパスワードを用いてダウンロードする。ダウンロード後、サーバーのデータは復元不可能な状態に消去する。なお、データを消去するまでの間に、サンスター株式会社の担当者が、保守点検やデータ管理の課程でデータを閲覧する可能性があるが、データを操作することはできず、またデータの利用も行わない。

また、共同研究機関であるサンスター株式会社へは、解析後の結果のみを提示する。

1. 研究対象者に生じる負担並びに予測されるリスク及び利益
2. 負担・リスク

GUM PLAYを使用し、日常的にブラッシングを実施することにより、時間的負担が生じる。

GUM PLAY使用中は、アプリをインストールし、使用することにより、インターネット利用料金が発生する。

1. 利益

ブラッシングの習慣化を促し、将来的な歯科疾患予防につながる可能性がある。

1. 負担・リスクと利益の総合的評価・最小化する対策

事前の文書や広報等において試験内容や調査内容、所要時間等を説明し、自由意志による参加ならびに、参加同意の撤回の機会を設けることを周知徹底する。また、金銭的な負担に関しては、謝礼や試験終了後のGUM PLAYの提供によって対策を行う。

1. 試料・情報の保管及び破棄の方法
2. 試料・情報等の保管期間

研究で得た試料・情報は、研究最終結果を報告した日から少なくとも10年間、保管する。

1. 試料・情報等の保管方法

研究で得た試料・情報は、京都大学環境安全保健機構健康科学センターの研究室（電子キーによる施錠可）において、施錠した机の中に保管し、漏えい、混交、盗難、紛失等が起こらないよう管理する。

1. 研究終了後に破棄する場合その処理方法

10年間が経過した後、紙媒体のデータはシュレッダーにて廃棄し、電子媒体のデータは復元不可能な状態にする。

1. 試料・情報の二次利用及び他の研究機関への提供の可能性

なし

1. 研究機関の長への報告及び方法
2. 研究の倫理的妥当性や科学的合理性を損なう事実もしくは情報、または損なうおそれのある情報を得た場合は、速やかに安全性情報に関する報告を行う。
3. 研究実施の適正性や研究結果の信頼性を損なう事実もしくは情報、または損なうおそれのある情報を得た場合は、速やかに不適合等報告書を提出する。
4. 年次報告は1回/3年以上実施する。中止・終了報告は適宜行う。
5. 研究の資金・利益相反
6. 研究資金の種類および提供者

共同研究費（提供元：サンスター株式会社）

1. 提供者と研究者との関係

共同研究者

1. 利益相反

本研究は、サンスター株式会社との共同研究費を資金源として実施する。研究者等と、本研究の試験機器として使用するGUM PLAYの製造企業であるサンスター株式会社との間において、利益相反に該当する事項はなく、GUM PLAYの提供をうけて研究を実施するが、それ以外の利害関係はない。研究の実施および成果に関して利益相反が生じる可能性があるため、京都大学の利益相反マネジメントポリシーに基づき、本研究実施前に利益相反審査委員会の審査および承認を受ける。

1. 研究対象者及びその関係者からの相談等への対応
2. 研究窓口

京都大学大学院医学研究科　社会健康医学系専攻　予防医療学分野

研究実施担当者：志田瑶

(E-mail) hamigaki_rct2018@hc2.hoken.kyoto-u.ac.jp

1. 京都大学相談窓口

京都大学医学研究科 総務企画課　研究推進掛

(Tel)　075-753-9301　(E-mail)　kikaku06@mail2.adm.kyoto-u.ac.jp

京都大学施設部環境安全保健課機構事務掛

（E-mail）810kikochosei@mail2.adm.kyoto-u.ac.jp

1. 研究対象者の経済的負担又は謝礼

- 2週目、4週目のすべての測定を終了した者には、5000円のQUOカード（実働1時間あたり1000円程度）を謝礼として進呈する。(加速度・振幅を測定した一部の対象者には6000円分のQUOカードを進呈する。)
- 試験終了後にGUM PLAYを提供する。

1. 研究対象者に係る研究結果（偶発的所見を含む）の取り扱い

本研究によって、研究対象者の口腔内診査を実施した際に、炎症等といった偶発的所見を得る可能性がある。歯科受診を勧めるような状態である場合は、歯科衛生士よりその旨を対象者に伝える。なお、子孫に受け継がれ得る遺伝的特徴などに関する重要な知見が得られる可能性はない。

1. 研究の実施体制
2. 研究責任者（研究の統括）

氏名：石見　拓

所属・職位：京都大学　環境安全保健機構 附属健康科学センター・教授

連絡先住所：京都市左京区吉田本町

電話番号：075-753-2426

E-mail ：iwami.taku.8w@kyoto-u.ac.jp

1. 研究実施者

①氏名：志田　瑶（プロトコール作成・運営・解析・論文執筆）

所属：京都大学大学院医学研究科　社会健康医学系専攻 専門職学位課程

連絡先住所：京都府京都市左京区吉田近衛町

②氏名：岡林　里枝（プロトコール作成・運営・解析・論文執筆）

所属・職位：京都大学 環境安全保健機構 附属健康科学センター・助教

連絡先住所：京都市左京区吉田本町

③氏名：川村　孝（プロトコール作成助言・論文執筆指導）

所属・職位：京都大学 環境安全保健機構 附属健康科学センター・教授

連絡先住所：京都市左京区吉田本町

1. 研究協力者

①氏名：吉岡　昌美（プロトコール作成助言）

所属・職位：徳島文理大学　保健福祉学部　口腔保健学科・教授

②氏名：清原　康介（統計解析責任者）

所属・職位：大妻女子大学　家政学部食物学科・専任講師

③氏名：小林　大介（割り付け担当）

所属・職位：京都大学 環境安全保健機構 附属健康科学センター・助教

④氏名：北村　哲久（効果安全性評価委員）

所属・職位：大阪大学大学院医学系研究科　社会医学講座　環境医学・助教

⑤氏名：松山　匡（効果安全性評価委員）

所属・職位：京都府立医科大学　救急医療学講座・助教

⑥氏名：西山　知佳（効果安全性評価委員）

所属・職位：京都大学大学院医学研究科　人間健康科学系専攻　臨床看護学講座　クリティカルケア看護学分野・講師

1. 共同研究者

①氏名：高世　尚子（プロトコール作成・運営）

所属・職位：サンスター株式会社　オーラルケアカンパニー兼ヘルス＆ビューティーカンパニー事業計画部・課長

連絡先住所：大阪市中央区今橋1-3-3

②氏名：岡澤　悠衣（プロトコール作成・運営）

所属・職位：サンスター株式会社　オーラルケアカンパニー　日本ブロック研究開発部　プロフェッショナルリレーショングループ・研究員

連絡先住所：大阪府高槻市上土室5-30-1

③氏名：西浦　正洋（プロトコール作成・運営）

所属・職位：サンスター株式会社　オーラルケアカンパニー兼ヘルス＆ビューティーカンパニー　アジア/日本ブロック事業サポート部　エレクトロメカ開発グループ・グループ長

連絡先住所：大阪府高槻市朝日町3-1

1. 氏名：松富　信治（プロトコール作成・運営）

所属・職位：サンスター株式会社　オーラルケアカンパニー　日本ブロック西日本支社九州支店　九州営業所兼新規チャネル開発室

連絡先住所：福岡県福岡市博多区店屋町8−24

1. 研究協力機関名

一般財団法人サンスター財団、徳島文理大学、大妻女子大学

1. 研究事業務の委託

なし

1. 研究実施計画書の変更、及び改訂

予想外の事態が生じた際には、研究実施計画書の変更を要する場合がある。その際は、研究実施メンバーが十分な協議の上で、修正・変更した研究実施計画書を京都大学大学院医学研究科・医学部及び医学部附属病院医の倫理委員会へ再申請を行う。

1. 遵守すべき倫理指針

研究者は世界医師会ヘルシンキ宣言を遵守し、個人情報保護法及び、「人を対象とする医学系研究に関する倫理指針」にのっとり研究対象者の個人を尊重し、人権を守る。

1. 研究成果の帰属とその公表

京都大学に帰属する。実施責任者は、大学内や専門の学会、学術雑誌での研究成果の論文投稿及び公表について責任をもつ。

1. 研究に関する情報公開の方法

大学病院医療情報ネットワーク（University Hospital Medical Information Network; UMIN）のUMIN臨床試験登録システムに事前登録を行う。（試験ID：UMIN000034503）

1. 参考文献
2. 労働省. 厚生労働省(2016)平成28年歯科疾患実態調査. 2016
3. 日本歯周病学会. 歯周病治療の指針2015. 2016
4. Khader YS．Periodontal diseases and the risk of coronary heart and cerebrovascular diseases: a meta-analysis. J Periodontol. 2004;75(8):1046-53.
5. Beck J, Garcia R, Heiss G, Vokonas PS Offenbacher S. Periodontal disease and cardiovasculardisease. J. Periodontol. 1996;67:1123-1137.
6. Grau AJ, Becher H, Ziegler CM, Lichy C, Buggle F, Kaiser C, Lutz R, Bultmann S, Preusch M Dorfer CE. Periodontal disease as a risk factor for ischemic stroke. Stroke. 2004;35:496-501.
7. 財団法人8020推進委員会. 歯周病と生活習慣病の関係. 2005 Mar.
8. Pace C. C, McCullough G.H. The association between oral microorganisms and aspiration pneumonia in the institutionalized elderly: review and recommendations. Dysphagia. 2010; 25:307-322.
9. Crocombe LA, Brennan DS, Slade GD, Loc DO. Is self interdental cleaning associated with dental plaque levels, dental calculus, gingivitis and periodontal disease? J Periodont Res. 2012;47:188–197.
10. Poyato-Ferrera M1, Segura-Egea JJ, Bullón-Fernández P. Comparison of modified Bass technique with normal toothbrushing practices for efficacy in supragingival plaque removal. Int J Dent Hyg. 2003;1(2):110-114.
11. Ganss C1, Schlueter N, Preiss S, Klimek J. Tooth brushing habits in uninstructed adults--frequency, technique, duration and force. Clin Oral Investig. 2009;13(2):203-208.
12. Wainwright J, Sheiham A. An analysis of methods of toothbrushing recommended by dental associations, toothpaste and toothbrush companies and in dental texts. Br Dent J. 2014;217(3):E5.
13. Schlueter N, Klimek J, Saleschke G, Ganss C. Adoption of a toothbrushing technique: a controlled, randomised clinical trial. Clin Oral Investig. 2010;14(1):99-106.
14. Creeth JE, Gallagher A, Sowinski J, Bowman J, Barrett K, Lowe S, Patel K, Bosma ML. The Effect of Brushing Time and Dentifrice on Dental Plaque Removal in vivo. J Dent Hyg. 2009;83(3):111-116.
